# Plasma Lactate Levels Increase during Hyperinsulinemic Euglycemic Clamp and Oral Glucose Tolerance Test

**DOI:** 10.1155/2015/102054

**Published:** 2015-04-19

**Authors:** Feven Berhane, Alemu Fite, Nour Daboul, Wissam Al-Janabi, Zaher Msallaty, Michael Caruso, Monique K. Lewis, Zhengping Yi, Michael P. Diamond, Abdul-Badi Abou-Samra, Berhane Seyoum

**Affiliations:** ^1^Wayne State University School of Medicine, Detroit, MI 48201, USA; ^2^Georgia Regents University, Augusta, GA 30912, USA; ^3^Hamad Medical Corporation, Doha, Qatar

## Abstract

Insulin resistance, which plays a central role in the pathogenesis of type 2 diabetes (T2D), is an early indicator that heralds the occurrence of T2D. It is imperative to understand the metabolic changes that occur at the cellular level in the early stages of insulin resistance. The objective of this study was to determine the pattern of circulating lactate levels during oral glucose tolerance test (OGTT) and hyperinsulinemic euglycemic clamp (HIEC) study in normal nondiabetic subjects. Lactate and glycerol were determined every 30 minutes during OGTT and HIEC on 22 participants. Lactate progressively increased throughout the HIEC study period (*P* < 0.001). Participants with BMI < 30 had significantly higher mean *M*-values compared to those with BMI ≥ 30 at baseline (*P* < 0.05). This trend also continued throughout the OGTT. In addition, those with impaired glucose tolerance test (IGT) had significantly higher mean lactate levels compared to those with normal glucose tolerance (*P* < 0.001). In conclusion, we found that lactate increased during HIEC study, which is a state of hyperinsulinemia similar to the metabolic milieu seen during the early stages in the development of T2D.

## 1. Introduction

Type 2 diabetes mellitus (T2D) is a serious global problem that is closely related to the rise in obesity. Insulin resistance plays a central role in the pathogenesis of T2D [1] and is an early marker for the disease. Therefore, it is imperative to have a simple method to identify insulin resistance in the early stages in order to implement preventative measures. The gold standard test for insulin resistance is the hyperinsulinemic euglycemic clamp (HIEC); however, this technique is cumbersome and is restricted for research. Other methods, such as the minimal model approximation of the metabolism of glucose (MMAMG) [2, 3] and homeostasis model assessment to estimate insulin resistance (HOMA-IR), can be also used; however, they are indirect derivatives computed from fasting insulin and glucose determinations.

Not much work has been done to understand the early metabolic changes at the cellular level during the stages of insulin resistance in the pathogenesis of diabetes. Understanding the early changes could help identify insulin resistance before it is clinically apparent. Part of the metabolic changes that occur at the cellular level is the increase in lactate production. In epidemiologic studies, it has been reported that lactate could predict the occurrence of diabetes [4, 5]. The purpose of this study is to examine lactate changes during HIEC and OGTT. HIEC is a state of hyperinsulinemia artificially created that resembles the hyperinsulinemia seen during the early stages of insulin resistance.

**Figure 1:**
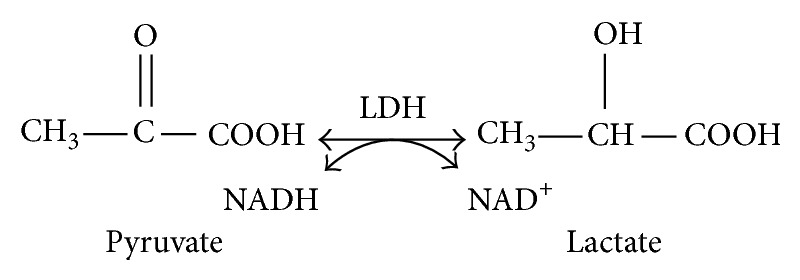
Generation of NAD^+^ from pyruvate.

Insulin stimulates glycolysis by activating the rate limiting enzymes, phosphofructokinase and pyruvate dehydrogenase [6]. Patients with diabetes and insulin resistance have increased activity of glycolysis [7, 8]. The increase in glycolysis leads to increased production of NADH and pyruvate and decreased NAD^+^ levels. NAD^+^ is generated from NADH in a redox reaction when pyruvate is converted to lactate by lactate dehydrogenase (Figure 1). This reaction might be exaggerated in insulin resistance as there is increased glycolysis driven by hyperinsulinemia. The conversion of pyruvate to lactate partly replenishes NAD^+^. Lactate is an important cellular metabolite in the glycolytic pathway and it may reflect the state of the cellular metabolism. Its high concentration may be an early signal of the beginning of insulin resistance. The purpose of this study was to examine lactate levels during OGTT and HIEC.

## 2. Methods

### 2.1. Study Subjects

A total of 22 healthy nondiabetic volunteers (16 males and 6 females) were included in this study. The mean age was 41 ± 12.4 years and the average BMI was 27.8 ± 4.8 kg/m^2^. Participants were 1st screened over the phone and provided a brief explanation of the study. Prequalified subjects were scheduled for the on-site screening visit. All studies were conducted in the MOTT Clinical Research Center (MCRC) on the Wayne State University medical campus and began at approximately 08:30 (time −60 min) after a minimum 10-hour overnight fast. The purpose, nature, and potential risks of the study were explained to all participants, and written consent was obtained before their participation. After written informed consent was obtained, comprehensive screening tests were performed such as vitals, body mass index (BMI), urinalysis, pregnancy test (females only), ECG, body composition, medical/health history, international physical activity questionnaire, and complete blood chemistry, CBC, HbA1c, and lipid profile. None of the participants had reported medical problems, and none of them was engaged in any heavy exercise. All participants were instructed to stop any form of exercise for at least 2 days before the study. The institutional review board of Wayne State University approved the protocol.

### 2.2. Experimental Strategy

*OGTT*. Patients reported to the Mott Clinical Research Center (MCRC) at 8:00 AM in a fasting state. A catheter was placed in their antecubital vein for repeated blood draws, which was covered with a heating pad (60°C) for sampling of arterialized venous blood. The IV line was kept open with infusion of normal saline (0.9% NaCl; pH 7.4). Baseline blood for glucose, insulin, and lactate measurements were drown at 0 time. Then patients were given 300 mL of an aqueous solution containing 75 grams of glucose with orange flavor to drink it at one time. Glucose, lactate, and glycerol concentrations were determined at 30 min intervals for 2 hours after glucose ingestion. Glucose was measured using a YSI glucose analyzer (Beckman Instruments, Fullerton, California, USA).

*Hyperinsulinemic Euglycemic Clamp (HIEC)*. The clamp study was performed at the MCRC. Subjects were admitted to the MCRC at 8:00 AM after an overnight fast for the hyperinsulinemic euglycemic clamp. An antecubital catheter was placed on the right hand for repeated blood draws, which was covered with a heating pad (60°C) for sampling of arterialized venous blood. The IV line was kept open with infusion of normal saline (0.9% NaCl; pH 7.4).

A second antecubital catheter with ports for insulin and glucose was inserted on the left arm. Continuous infusion of human regular insulin (Humulin R; Eli Lilly, Indianapolis, IN, USA) was started at a rate of 80 mU m^−2^ minute^−1^ and continued for 120 minutes. Plasma glucose was measured with an YSI glucose analyzer at 5-minute intervals throughout the hyperinsulinemic euglycemic clamp. Euglycemia was targeted for 90 mg/dL by variable infusion of 20% D-glucose [9]. Insulin-stimulated glucose disposal rates (*M*-value) were calculated as the average value during the final 30 minutes of insulin infusion. *M*-value is the glucose infusion rate per kg per minute (mg/kg/min).

Lactate and glycerol levels were determined before the initiation of insulin infusion at −30 minutes and then every 30 minutes during the hyperinsulinemic conditions (0, 30, 60, 90, and 120 minutes).

*Lactate Assay*. Fasting plasma lactate levels were determined using the commercially available enzymatic kit (Eton Bioscience Inc., San Diego, CA). Lactate determination technique relies on the lactate dehydrogenase conversion of lactate to pyruvate. In the process NAD^+^ is reduced to NADH that is ultimately coupled with the tetrazolium salt 2-(4-iodophenyl)-3-(4-nitrophenyl)-5-phenyl tetrazolium (INT) to produce a formazan, which exhibits absorbance at 490 nm. The assay is specific for lactate and the intraassay and interassay variations are 6% and 15%, respectively. As compared to other studies [5, 10, 11] this method showed a better precision. Coefficient of determination computed from lactate standard curve was 0.996.

*Glycerol Assay*. Cayman's Assay kit was used for plasma glycerol determination (Cayman Chemical Company, Ann Arbor, MI, USA). The assay employs a coupled enzymatic reaction system in 96-well plates whereby plasma glycerol content is converted by glycerol kinase. The enzymatic reaction produces glycerol-3-phosphate (G3P) in the presence of adenosine-5′-diphosphate (ADP). The G3P is oxidized by glycerol phosphate oxidase producing dihydroxyacetone phosphate and hydrogen peroxide (H_2_O_2_). The H_2_O_2_ reacts with 4-aminoantipyrine (4-AAP) and N-ethyl-N-(3-sulfopropyl)-m-anisidine (ESPA) by the catalysis of horse radish peroxidase (HRP). Briefly, 10 *μ*L of undiluted plasma was added to the plates. Reaction was initiated by adding a total of 150 *μ*L of buffer and solutions of enzyme mixture provided with the kit. After appropriate incubation period, the final colored product was read using a spectrophotometer (Molecular Devices LLC, CA, USA) at an absorbance maxima of *λ*_540 nm_. Final concentration of plasma glycerol is determined based upon a standard curve of known glycerol concentration series run with each analysis.

### 2.3. Statistical Analysis

We used SPSS statistical software (SPSS Inc., Chicago, IL) to calculate significant differences in lactate, glycerol levels, OGTT, and *M*-values. We also performed statistical correlations between these variables, in addition to BMI, considering all the subjects as one population to examine for trends and positive and negative correlation among these variables. We analyzed whether or not there was a rise in lactate or glycerol over time for both OGTT and HIEC. Then, we grouped the participants according to those with normal and impaired OGTT. Lastly, we grouped the participants into those with *M*-values ≤ 4 and those with *M*-values > 4. We compared the overall mean lactate levels for these two groups during OGTT and HIEC. These analyses allowed us to examine our hypothesis that lactate increases in individuals with clinical parameters suggestive of insulin resistance (IGTT, low *M*-values).

## 3. Results

There were a total of 22 subjects: 16 were males and 6 were females. The mean age was 41.9 ± 2.6 years (SEM) ranging 20–59 years and the mean BMI was 27.7 ± 1.0 kg/m^2^.

### 3.1. OGTT and HIEC

All subjects were able to complete both OGTT and HIEC without any difficulties. The mean fasting blood glucose during OGTT was 86.6 ± 1.6 mg/dL and the entire mean *M*-value during HIEC was 8.2 ± 1.0. There was no correlation between fasting blood glucose and *M*-values. Seven participants (31.8%) were found to have impaired OGTT (IGT).

### 3.2. Lactate and Glycerol during HIEC

**Figure 2:**
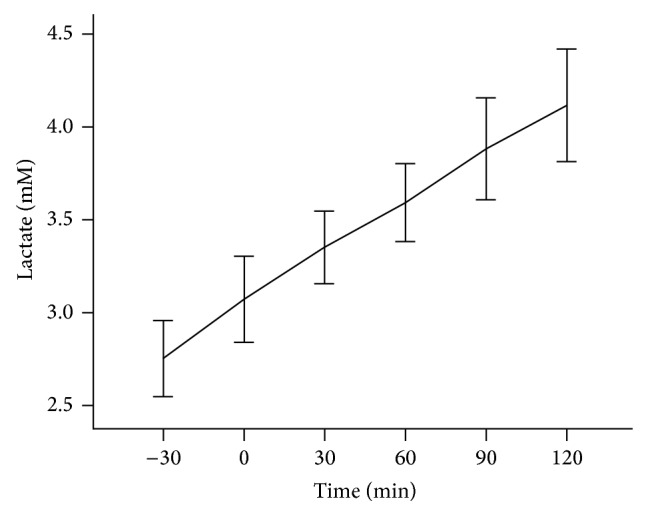
Lactate levels during HIEC. Plasma lactate was measured as shown in Study Subjects. The figure shows lactate levels increase over time.

During HIEC there was a linear rise in plasma lactate levels. It increased significantly and progressively over the entire duration of the clamp time (*P* < 0.001, Figure 2). The progressive increase in lactate during HIEC was seen on all participants. There was no statistically significant correlation with the *M*-values, neither was there any relationship when *M*-values were divided by quartiles. However when the *M*-values were divided above and below 10, the mean lactate level at 0 time, which is the time when HIEC was initiated, the mean lactate level was significantly elevated among those participants with *M*-values below 10, (*P* < 0.03).

**Figure 3:**
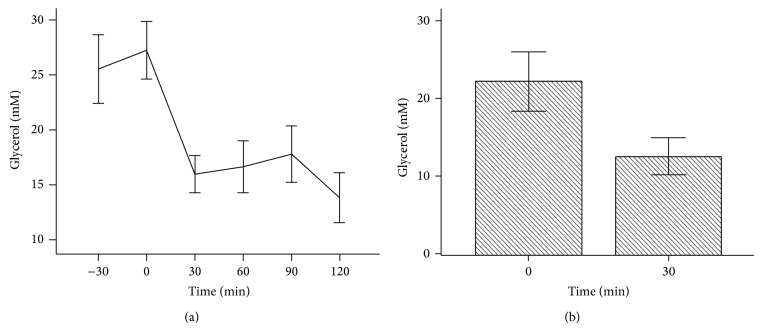
Glycerol levels during HIEC. Plasma glycerol was measured as shown in Study Subjects. (a) showed glycerol levels drop between time 0 min and 30 min and then stayed steady. (b) showed glycerol at time 30 min is significantly lower than glycerol levels at time 0 min (*P* < 0.001).

The level of plasma glycerol was high before the initiation of clamp that is at time −30 (30 minutes before HIEC started) and remained high till time 0, the time when HIEC was initiated. Then it dropped precipitously and remained suppressed during the whole clamp period (Figure 3).

**Figure 4:**
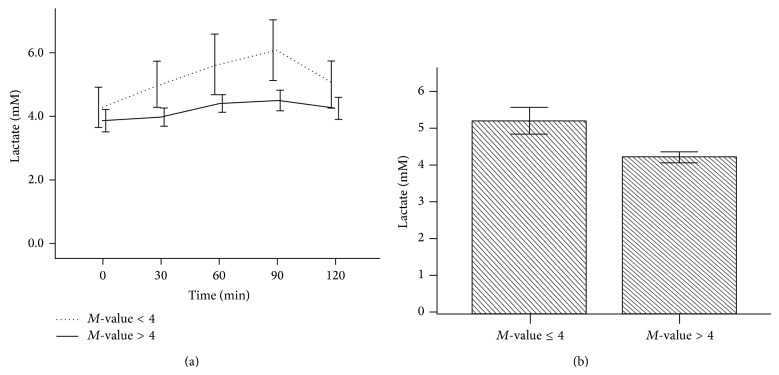
Plasma lactate values from OGTT according to *M*-value. OGTT was based on 75 g oral glucose tolerance tested over 120 min. *M*-values represent the insulin resistance index at time 120 min of HIEC. Plasma lactate was measured as shown in Study Subjects. (a) showed a progressive increase in lactate during OGTT among patients with *M*-value ≤ 4. The difference is not statistically different but did show a trend. (b) showed the mean total lactate among subjects with *M*-value greater or less than 4. Participants having *M*-values above 4 had significantly lower lactate levels (*P* < 0.01).

During OGTT, those subjects with *M*-values ≤ 4 had slightly higher lactate values at baseline that were maintained throughout the period of OGTT than those with *M*-value > 4. The difference showed a strong trend but did reach statistical significance (Figure 4(a)). However, the mean total lactate among subjects with *M*-values less than 4 was significantly higher than those having *M*-values above 4 (5.2 ± 2.5 mM versus 4.2 ± 1.9 mM, *P* < 0.01) (Figure 4(b)).

**Figure 5:**
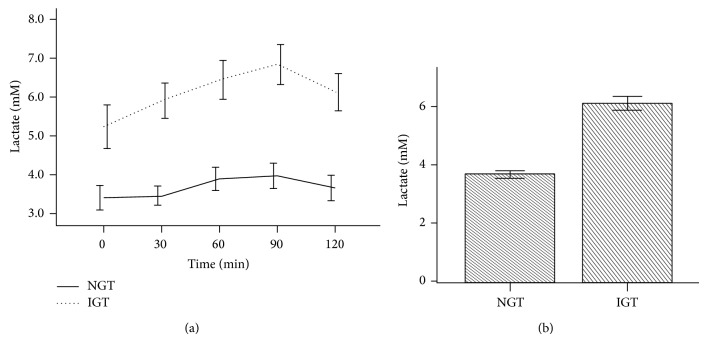
Lactate levels during OGTT according to glucose tolerance. OGTT was based on 75 g oral glucose tolerance tested over 120 min. Normal glucose tolerance (NGT) is based on glucose levels less than or equal to 140 mg/dL at time 120 min, and impaired glucose tolerance (IGT) is based on glucose levels 140–200 mg/dL at time 120 min. Plasma lactate was measured as shown in Study Subjects. (a) showed significantly higher levels of lactate among participants with IGT. The difference continued throughout the period of OGTT. (b) showed mean total lactate levels during OGTT. The cumulative lactate level among participants with IGT was significantly higher than those participants with NGT (*P* < 0.001).

Additionally, the fasting lactate level was significantly higher among patients with IGTT than NGTT. The difference continued during the entire period of OGTT. For those with IGTT the lactate level increased progressively whereas for those with NGTT the lactate level was stable throughout the test (Figure 5(a)). Likewise lactate was significantly higher among those with IGTT than those with NGTT (5.7 ± 2.0 mM versus 3.7 ± 1.7 mM, *P* < 0.001) (Figure 5(b)).

### 3.3. Lipid Profile

**Figure 6:**
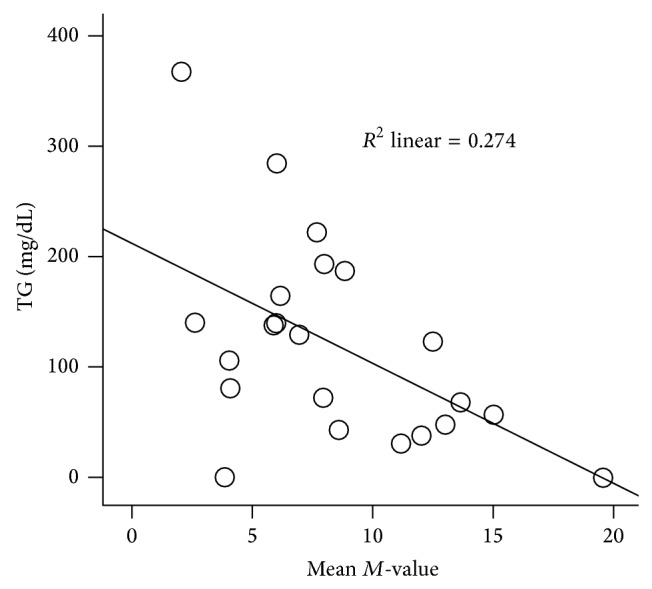
Correlation of triglycerides and mean *M*-values. Triglyceride levels negatively correlated with *M*-values.

The mean total cholesterol, LDL cholesterol, HDL cholesterol, and triglyceride levels were 183.7 ± 10.9, 102.7 ± 10.3, 49.6 ± 3.2, and 131.7 ± 19.6 mg/dL. There was no correlation between total LDL and *M*-values; however there was a strong negative correlation between triglycerides and *M*-values (*r* = −0.50, *P* = 0.01). Participants with lower *M*-value had higher triglycerides (Figure 6). On the contrary, with regard to HDL there was a trend for positive correlation. In addition, those with low *M*-values had low HDL. There was also strong negative correlation between total cholesterol and *M*-values (*r* = −0.50, *P* = 0.01).

## 4. Discussion

This study demonstrated for the first time that lactate production progressively and proportionally increases during the entire HIEC study in normal and obese subjects, a situation that mimics the hyperinsulinemic state seen in the early stages of diabetes before insulin resistance becomes clinically apparent and before the patient presents with diabetes. In similar previous studies, it is intriguing to observe increased lactate levels during the early periods of diabetes, prediabetes or the stage where there is hyperinsulinemia. Lactate is not only increased in the early stages of diabetes but has also been shown to predict its occurrence in the future [4, 5]. For the first time we have demonstrated a progressive increase in lactate and suppression of glycerol production during the entire period of HIEC. Furthermore, based on our study we cautiously state that lactate could be used to identify a state of insulin resistance.

**Figure 7:**
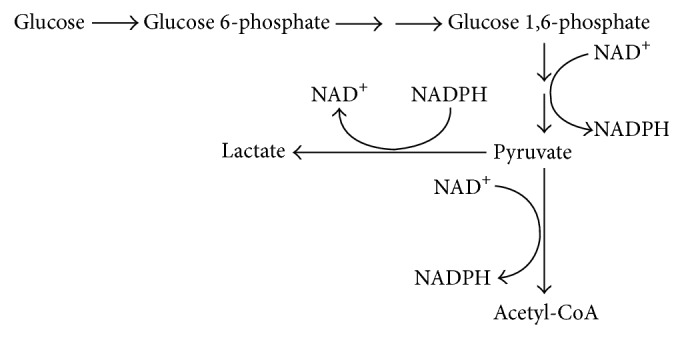
Consumption and regeneration of NAD^+^.

Traditionally an increase in lactate has been used as an indicator of energy imbalance related to vigorous exercise and hypoxia [12]. However, in our situation lactate is not increased as a result of exercise but it is increased in reaction to the hyperinsulinemic state that is artificially created by the HIEC. What will be the connecting tread between hyperinsulinemia and the increased lactate? A plausible explanation for the increased lactate levels during hyperinsulinemia could be an inadequate oxidative capacity at the cellular level. Indeed several studies have reported defective oxidative phosphorylation during the development of insulin resistance [8, 13, 14]. It is our belief that at this stage it is the defective oxidative capacity that causes the significant increase in circulating lactate. The hyperinsulinemic stage that we artificially created during the HIEC reflects the early stages of hyperinsulinemia that is seen during the pathogenesis of type 2 diabetes. It is very hard to conclude and the data we have is not able to confirm or refute that there is or there is no insulin resistance during the period of progressive lactate increase. But one can safely say that there is insulin resistance that goes along with increased insulin secretion without going into the age-old dilemma of which comes first the egg or the chicken. We cannot conclude in this study whether the insulin resistance or the hyperinsulinemia comes first. Nonetheless the end result is the high level of lactate, which is seen concurrently with hyperinsulinemia. The high levels of circulating insulin stimulate glycolysis, anaerobic glucose metabolism, producing excessive pyruvate that is further converted to lactate leading to high circulating lactate levels (Figure 7). Studies have shown that glycolysis increases in patients with diabetes [7, 8].

During the stage of hyperinsulinemia, the additional stimuli for the body to convert pyruvate to lactate, instead of converting it to acetyl CoA and sending it to the Krebs cycle, is the apparent defective oxidative capacity existing at the cellular level. This defect is manifested with a high NADH/NAD^+^ ratio. The increase in glycolysis activity depletes the cellular concentration of NAD^+^ and increases NADH concentration, tilting the balance toward a high NADH/NAD^+^ ratio. The resultant high NADH level creates a cellular reductive stress that drives the conversion of pyruvate to lactate, to quickly generate NAD^+^ (Figure 7). Moreover, the additional factor for the cell to recover NAD^+^ is in order to continue glycolysis. Adequate presence of NAD^+^ is mandatory for glycolysis to continue. Under normal conditions NAD^+^ is regenerated through Krebs cycle in the mitochondria. However, during the early stage of diabetes when there is hyperinsulinemia and excess calorie intake, the activity of glycolysis is increased. In this situation the cellular level of NAD^+^ is depleted, stimulating the cell mechanism to generate NAD^+^ quickly outside of the mitochondria. The fast metabolic pathway to generate NAD^+^ in the cytosol is by oxidizing NADH back to NAD^+^ through the conversion of pyruvate to lactate by lactate dehydrogenase (see Figure 1). Additionally the prevailing mitochondrial dysfunction seen in insulin resistance is a further impetus for the cell to generate NAD^+^ in the cytoplasm by converting pyruvate to lactate [14]. Thus the findings in this pilot study indicate that lactate is increased significantly in subjects with hyperinsulinemia, a condition that we can safely say is a good indicator of a state of insulin resistance.

Fasting lactate levels were significantly higher among patients with IGT than those with NGT and the gap widened with time (Figure 5). Robertson et al. [15] have also observed similar results. They showed a rise in lactate and insulin levels in subjects with IGT. The increase in lactate among patients with IGT was more clearly seen in an experiment done by Krentz et al. [16] who demonstrated significant elevation of lactate among healthy, nonobese subjects with IGT who were matched for age, gender, and body mass index. The subjects who had IGT and elevated lactate also had hyperinsulinemia despite a normal fasting glucose, a scenario commonly seen during the early stages of insulin resistance. Furthermore Robertson et al. [17] also demonstrated that lactate levels correlate positively with insulin levels: as insulin levels increase lactate concentration increased. Again it is demonstrated that lactate increases in a metabolic situation where there is insulin resistance. These findings are in line with our results that lactate rises during hyperinsulinemia. Additionally these findings support the hypothesis that an elevated lactate level may herald insulin resistance.

Along with the increase in lactate, different investigators have also demonstrated a similar increase in fatty acids [15–17], which is a common finding among patients with metabolic syndrome. One of the defining characteristics of metabolic syndrome is high triglycerides and low HDL. Similarly, we found significant negative correlation between triglycerides and *M*-values (Figure 6) with a trend showing a positive correlation with HDL. The positive correlation of lactate with triglycerides is an additional factor supporting our hypothesis that lactate increases in insulin resistance.

In conclusion, we demonstrated a linear rise of plasma lactate concentration during HIEC and IGT. The significance of the increased plasma lactate concentration during hyperinsulinemia may be relevant to the increased lactate levels in insulin resistant subjects with hyperinsulinemia; thus we suggest that an increase in lactate could herald the early stages of insulin resistance long time before patients are diagnosed with diabetes mellitus.
